# Whole-Genome Analysis of *Starmerella bacillaris* CC-PT4 against MRSA, a Non-*Saccharomyces* Yeast Isolated from Grape

**DOI:** 10.3390/jof8121255

**Published:** 2022-11-28

**Authors:** Yong Shen, Xue Bai, Xiran Zhou, Jiaxi Wang, Na Guo, Yanhong Deng

**Affiliations:** 1 College of Food Science and Engineering, Jilin University, Changchun 130062, China; 2 College of Veterinary Medicine, Jilin University, Changchun 130062, China

**Keywords:** *Starmerella bacillaris*, whole-genome, secondary metabolite, lysostaphin

## Abstract

*Starmerella bacillaris* is often isolated from environments associated with grape and winemaking. *S. bacillaris* has many beneficial properties, including the ability to improve the flavor of wine, the production of beneficial metabolites, and the ability to biocontrol. *S. bacillaris* CC-PT4 (CGMCC No. 23573) was isolated from grape and can inhibit methicillin-resistant *Staphylococcus aureus* and adaptability to harsh environments. In this paper, the whole genome of *S. bacillaris* CC-PT4 was sequenced and bioinformatics analyses were performed. The *S. bacillaris* CC-PT4 genome was finally assembled into five scaffolds with a genome size of 9.45 Mb and a GC content of 39.5%. It was predicted that the strain contained 4150 protein-coding genes, of which two genes encoded killer toxin and one gene encoded lysostaphin. It also contains genes encoding F1F0-ATPases, Na(+)/H(+) antiporter, cation/H(+) antiporter, ATP-dependent bile acid permease, major facilitator superfamily (MFS) antiporters, and stress response protein, which help *S. bacillaris* CC-PT4 adapt to bile, acid, and other stressful environments. Proteins related to flocculation and adhesion have also been identified in the *S. bacillaris* CC-PT4 genome. Predicted by antiSMASH, two secondary metabolite biosynthesis gene clusters were found, and the synthesized metabolites may have antimicrobial effects. Furthermore, *S. bacillaris* CC-PT4 carried genes associated with pathogenicity and drug resistance. Overall, the whole genome sequencing and analysis of *S. bacillaris* CC-PT4 in this study provide valuable information for understanding the biological characteristics and further development of this strain.

## 1. Introduction

Non-Saccharomyces *Starmerella bacillaris* was commonly found in grape, winemaking, and related environments [1,2]. The species was first isolated in botrytis-affected wine in Napa Valley (USA) in 2002 and was subsequently isolated from white wine in Zemplin, Hungary and identified and named *Candida zemplinina* as a novel species [3,4]. After isoenzyme profiles, 26S rDNA restriction profiles, and 26S rDNA sequencing analysis, it was finally classified as *S. bacillaris* [5].

*S. bacillaris* has good tolerance to high osmotolerance, which can tolerate high concentrations of sugar and ethanol, and this strain produces glycerol and low levels of ethanol and a variety of flavors during fermentation [4,6]. Furthermore, *S. bacillaris* has a fructophilic character and *Saccharomyces cerevisiae* has a glucophlilic character, which enables them to coexist for a long time in the fermentation process [7]. Many studies have found that the mixed fermentation of *S. bacillaris* with *S. cerevisiae* is beneficial to improve the aroma of wine [8,9,10].

Chemical fungicides were the traditional method to inhibit pathogens, but repeated use of these compounds usually leads to various adverse effects, such as drug resistance [11]. However, biocontrol agents are a potential alternative to reduce the use of chemical fungicides. *S. cerevisiae*, *S. boulardii,* and *Kluyveromyces marxianus* have all been shown to have biocontrol effects [12,13,14]. *S. bacillaris* has also been shown to have biocontrol potential to inhibit *Botrytis cinerea*, *Penicillium expansum,* and methicillin-resistant *Staphylococcus aureus* (MRSA) [15,16,17]. The possible antibacterial mechanisms of *S. bacillaris* include nutrient competition, space competition, production of degrading enzymes, killer toxin, and volatile organic compounds [15,18,19,20].

High-throughput whole-genome sequencing is an effective approach to gaining a more comprehensive understanding of the technological properties and safety of strains at the gene level. *S. bacillaris* has been shown to have important application potential, but the analysis of its genome has not been sufficient. Moreover, the genes of *S. bacillaris* isolated from different environments have high genetic diversity [21]. However, there are only data of five assembly genomes of *S. bacillaris* in NCBI (https://www.ncbi.nlm.nih.gov/, accessed on 26 October 2022). In 2017, the first *S. bacillaris* whose genome was sequenced was *S. bacillaris* FRI751, with a genome size of 9.3 Mb and a GC content of 39.4% [22]. For better development and application of *S. bacillaris*, it is necessary to sequence and analyze more *S. bacillaris* strains. Therefore, the purpose of this study was to sequence the genome of *S. bacillaris* CC-PT4 isolated from grape with anti-MRSA ability based on Illumina NovSeq sequencing platform and PacBio Sequel sequencing platform and to analyze the beneficial properties, secondary metabolites, biosynthesis gene cluster, killer toxin, lysostaphin, adaptability to stress, flocculation, and adhesion safety. 

## 2. Materials and Methods

### 2.1. Yeast Strain and Growth Conditions

The strain *S. bacillaris* CC-PT4 (CGMCC No. 23573) was isolated from grapes in our previous study [17]. *S. bacillaris* CC-PT4 was inoculated in a 250 mL flask containing 100 mL yeast peptone dextrose broth medium and cultured at 30 °C for 24 h. Afterward, cell pellets were harvested by centrifugation at 3000 rpm for 10 min, followed by DNA extraction and sequencing.

### 2.2. Genome Sequencing and Assembly

The *S. bacillaris* CC-PT4 genome was sequenced by Illumina NovSeq sequencing platform (2 × 150 bp paired-end reads) and PacBio Sequel sequencing platform with continuous long read sequencing (Personalbio, Shanghai, China). After the sequencing, Falcon and CANU [23] software were used to assemble the sequencing data to construct contig and scaffold, and pilon v1.18 [24] software was used to correct the assembly. Finally, BUSCO (Benchmarking Universal Single-Copy Orthologs, v3.0.2) [25] was used to evaluate the integrity of genome assembly.

### 2.3. Genome Annotation and Analyses

After obtaining the assembly genome, tandem repeats and interspersed repeats were identified by RepeatModler and RepeatMasker [26]. In the analysis of non-coding RNA, tRNA was predicted by tRNAscan-SE [27], rRNA was predicted by RNAmmer1.2 [28], and the prediction of other non-coding RNAs was obtained by comparing with Rfam [29]. Protein-coding genes were predicted by Augustus (version 3.03) [30], glimmerHMM (version 3.0.1) [31], GeneMark-ES (version 4.35) [32], and exonerate (version 2.2.0), and the prediction results were integrated by EVidenceModeler (version r2012-06-25) [33].

Functional annotation of protein-coding genes was performed by searching against databases, including NCBI nr, eggNOG, KEGG, Swiss-Prot, GO, P450, TCDB, Pfam, PHI, CAZy, DFVF. The related genes involved in stress adaptation, killer toxin, lysostaphin, and drug resistance were searched in the annotation of *S. bacillaris* CC-PT4 genome. Secreted proteins and membrane proteins were predicted by SingalP and TMHMM. Secondary metabolite biosynthesis gene clusters in the *S. bacillaris* CC-PT4 genome were predicted using anti-SMASH [34], and the predicted gene clusters were annotated with the MIBIG database [35]. The *S. bacillaris* CC-PT4 flocculation- and adhesion-related genes were analyzed according to the reported method [36]. First, the flocculation- and adhesion-related protein sequences were downloaded from Saccharomyces Genome Database (SGD) and then *S. bacillaris* CC-PT4 genome and these sequences were subjected to BLAST analysis (e-value < 1 × 10^−5^, identity > 30%).

### 2.4. Nucleotide Sequence Accession Number

This Whole Genome Shotgun project has been deposited at DDBJ/ENA/GenBank under the accession JAPDUH000000000. The version described in this paper is version JAPDUH010000000.

## 3. Results

### 3.1. Genome Sequencing and Assembly

**Figure 1:**
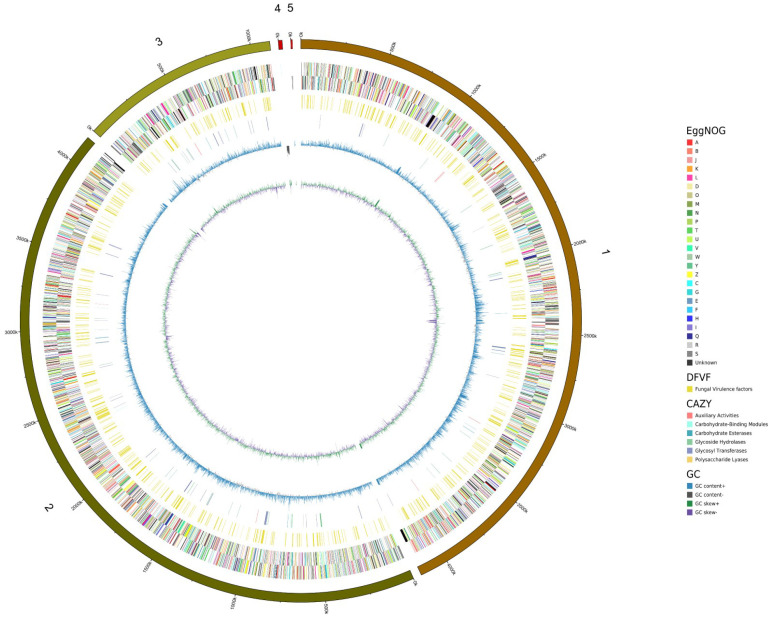
The circular graphical map of *S. bacillaris* CC-PT4 genome. From center to outside: the first circle represented GC skew, the second represented GC content, the third represented CAZy, the fourth represented DFVF, the fifth represented the COG regarding each CDS on the nonsense strand, the sixth represented the COG regarding each CDS on the sense strand, and seventh circles represented scale.

The *S. bacillaris* CC-PT4 whole-genome sequence was finally assembled into five scaffolds with a total length of 9451933 bp. The longest scaffold was 4159713 bp, the shortest scaffold was 8503 bp, N50 is 4154241 bp, and the GC content was 39.50% (Supplementary Table S1). The circular graphical map of the *S. bacillaris* CC-PT4 genome is shown in Figure 1.

In this study, BUSCO software was used to evaluate the integrity of the genome. The software predicts the integrity of genome assembly through the evolutionary information of single-copy direct homologous genes existing in all species in the fungal community. Finally, the integrity of genome assembly was assessed by the percentage of the number of single-copy genes that were completely aligned in the genome sequence to the total number of single-copy genes. In the *S. bacillaris* CC-PT4 genome, 63.3% of the genes were completely aligned to the BUSCO single-copy gene, and 33.0% were not mapped (Supplementary Table S2).

### 3.2. Genomic Functional Element Profiling

It was predicted that the strain *S. bacillaris* CC-PT4 had 286 tandem repeats and 1020 interspersed repeats scattered in the genome (Supplementary Table S3). The predicted results of non-coding RNAs in the *S. bacillaris* CC-PT4 genome were shown in Supplementary Table S4. Non-coding RNA (ncRNA) mainly includes tRNA, rRNA, snoRNA, microRNA, siRNA, snRNA, exRNA, piRNA, scaRNA, and lncRNA. *S. bacillaris* CC-PT4 had one-hundred-twenty-three tRNAs, four rRNAs, and seven other non-coding RNAs. It was predicted that the strain *S. bacillaris* CC-PT4 had 4150 genes found in the genome, accounting for 63.70% of the total genome length, and the average length of each gene is 1450.8 bp. There were 4266 exons, with an average of one exon per gene, accounting for 63.44% of the total genome length (Supplementary Table S5).

### 3.3. Gene Annotation

In this study, protein-coding genes were annotated using several databases. Among protein-coding genes, 4056 (97.73%) genes were annotated in the P450 database, followed by the NCBI nr database (3538, 5.25%), eggNOG database (3185, 76.45%), KEGG database (2610, 62.89%), Swiss-Prot database (3363, 81.04%), Go database annotations (2888, 69.59%), and TCDB database (670, 16.14%).

**Figure 2:**
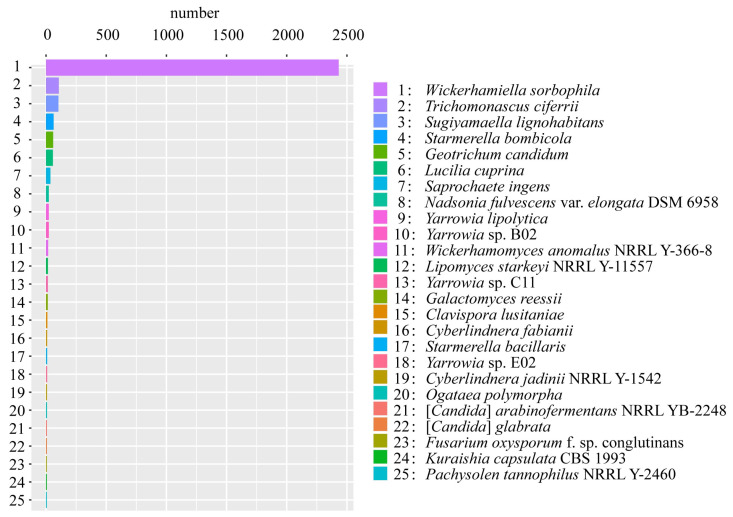
Species information statistics of *S.bacillaris* CC-PT4 genome annotated in NCBI nr (top 25 species).

The NCBI nr database is a non-redundant protein database. The goal is to provide a comprehensive dataset representing complete sequence information of any species [37]. Annotation results of this database contain species information. In this study, the species information of 3538 genes annotated by *S. bacillaris* CC-PT4 in NCBI nr was counted, and the top 25 species involved 3074 genes (Figure 2). However, ten genes whose sequence identity was not less than 97% among the annotated genes belong to the genus *Starmerella*, and nine genes are *S. bacillaris*, of which four genes had 100% sequence identity (Table 1).

**Figure 3:**
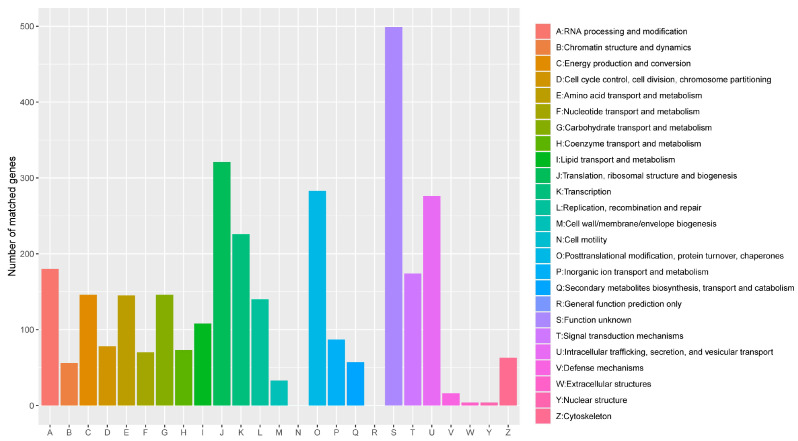
eggNOG functional classification diagram.

Further, eggNOG was used to annotate the function of *S. bacillaris* CC-PT4 annotated protein, and cluster analysis was carried out. The classification results of 3185 genes annotated by eggNOG on *S. bacillaris* CC-PT4 were shown in Figure 3. There were 499 genes with no clear function, which may be related to the lack of research on *S. bacillaris* and the lack of reference genes. The most abundant annotated genes with clear functional classification were translation, ribosomal structure, and biogenesis (321 genes); and genes related to carbohydrate transport and metabolism, lipid transport and metabolism, and amino acid transport and metabolism are 146, 108, 145, respectively. However, the information annotated by extracellular structures and nuclear structures was less (four genes). There were 57 genes related to secondary metabolites biosynthesis, transport, and catabolism. In addition, no cell-motility-related genes were found, which was consistent with the fact that yeast is a non-motile microorganism [38].

**Figure 4:**
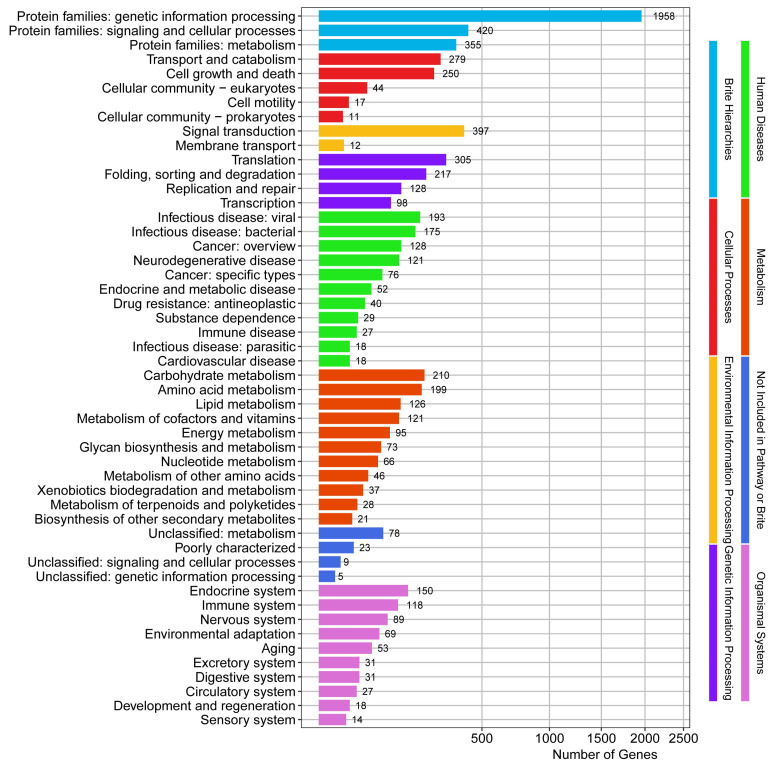
KEGG classification of *S. bacillaris* CC-PT4 genome.

The *S. bacillaris* CC-PT4 genome had 2610 genes annotated in the KEGG, which were divided into eight categories and fifty subcategories. The results are shown in Figure 4. Among them, the genes related to function of genetic information processing were the most abundant, followed by signaling and cellular processes.

Each entry in the Swiss-Prot database has detailed annotations. All sequence entries have been carefully verified by experienced molecular biologists and protein chemists through computer tools and reviewing relevant literature. The number of annotations of the *S. bacillaris* CC-PT4 genome in the Swiss-Prot database was 3363.

**Figure 5:**
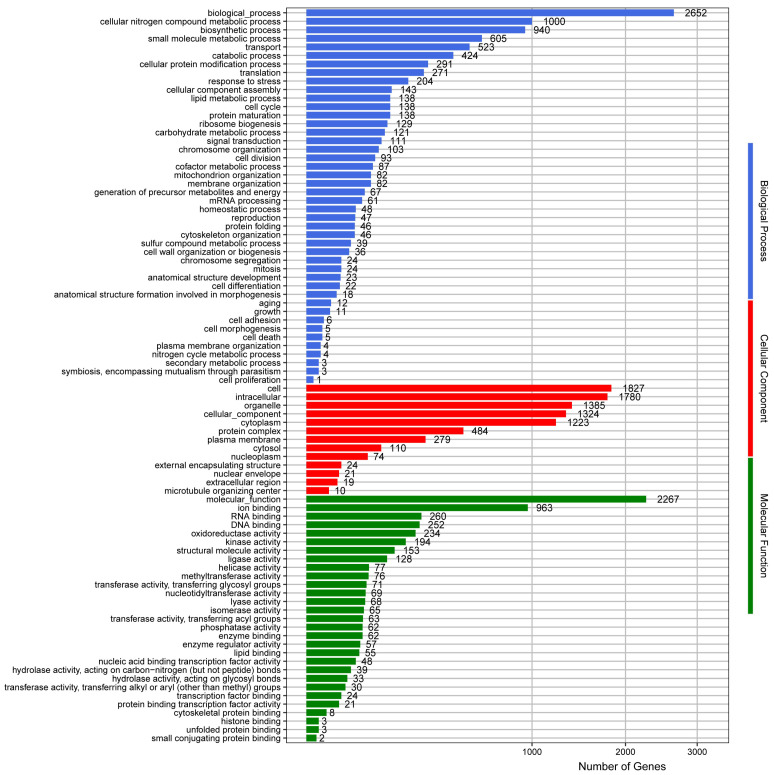
GO annotation of *S. bacillaris* CC-PT4 genome.

The GO database divides gene functions into molecular function, cellular component, and biological process. At the same time, a gene can be annotated multiple times by GO terms. The results of the *S. bacillaris* CC-PT4 genome annotated by the GO database were shown in Figure 5. The *S. bacillaris* CC-PT4 genome had 4109 annotated genes for molecular function, 8560 for cellular component, and 10,108 for biological process in the GO database.

### 3.4. CAZy Analysis

The CAZy database mainly contains a family of enzymes related to degradation, modification, and generation of glycosidic bonds. The family of enzymes was divided into six categories: glycoside hydrolases (GHs), polysaccharide lyases (PLs), carbohydrate esterases (CEs), glycosyl transferases (GTs), auxiliary activities (AAs), and carbohydrate-binding modules (CBM). It was predicted that *S. bacillaris* CC-PT4 contains 95 CAZy enzyme genes (Table 2).

### 3.5. Secondary Metabolite Biosynthesis Gene Clusters

**Figure 6:**
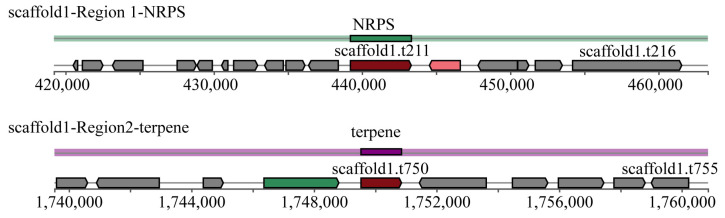
Predicted results of secondary metabolite biosynthesis gene clusters in the *Starmerella bacillaris* CC-PT4.

Two secondary metabolite biosynthesis gene clusters were found in the *S. bacillaris* CC-PT4 genome predicted by antiSMASH (Figure 6). They were the non-ribosomal peptide synthetase cluster (NRPS) and the terpene class, respectively. The above genes were annotated with the MIBIG database, and the annotation results with the highest BLAST score for each gene were shown in Table 3.

### 3.6. Killer Toxin and Lysostaphin Encoding Genes

Killer toxin is a toxic protein secreted by yeast that can kill other yeasts but has no killing effect on itself [39]. Lysostaphin can specifically hydrolyze the pentaglycine crosslinks of *S. aureus* peptidoglycan, resulting in lysis of *S. aureus*, and has the same bacteriostatic effect on drug-resistant *S. aureus* [40]. According to the annotation results of the *S. bacillaris* CC-PT4 genome, two genes encoded killer toxins and one gene encoded lysostaphin (Table 4).

### 3.7. Adaptation to Stress Analysis

According to the annotation of the yeast genome in eggNOG, Swiss-Prot, and the NCBI nr database, the related genes involved in the stress adaptation were searched. The results showed that there were many genes in the *S. bacillaris* CC-PT4 genome that facilitate strain adaptation to harsh stress, including pH stress resistance, bile stress resistance, oxidative stress resistance, ionic and heavy metal stress resistance, heat stress resistance, and other stress resistance (Supplementary Table S6).

### 3.8. Flocculation and Adhesion Analysis

**Figure 7:**
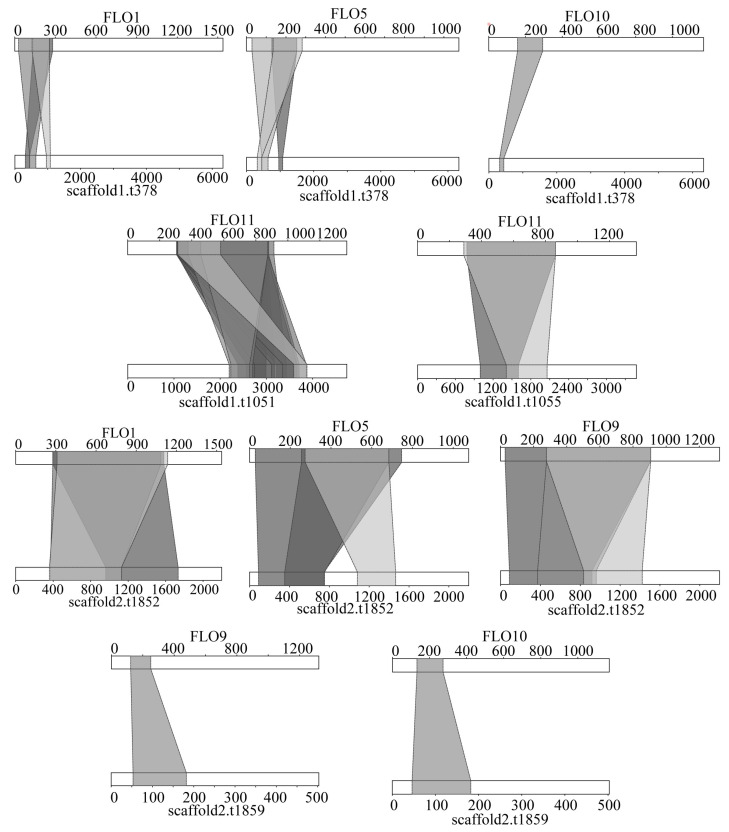
BLAST analysis *S. bacillaris* CC-PT4 genome against the flocculation protein sequence.

The protein sequences of FLO1, FLO5, FLO8, FLO9, FLO10, FLO11, FIG2, and AGA1 encoding flocculation were downloaded from SGD and subjected to BLAST against the *S. bacillaris* CC-PT4 genome. The results showed that five genes (scaffold1.378, scaffold1.1051, scaffold1.1055, scaffold2.1852, scaffold2.1859) in the *S. bacillaris* CC-PT4 genome encoded flocculation proteins (Figure 7, Supplementary Table S7). It can be seen from the Figure 7 that gene scaffold1.378 had sequence identity with three flocculation proteins, FLO1, FLO5, and FLO10, gene scaffold2.1852 had sequence identity with FLO1, FLO5, and FLO9, and gene scaffold2.1859 had sequence identity with FLO9 and FLO10. In addition, these genes share identity with multiple sequence fragments of flocculation protein.

Further, 41 protein sequences related to adhesion were downloaded from SGD, and the *S. bacillaris* CC-PT4 genome and these proteins were analyzed by BLAST. The results showed that 65 genes in the *S. bacillaris* CC-PT4 genome had sequence identity with one or more proteins of adhesion protein BGL2, CKA2, CRH1, CRR1, CWH41, DCW1, DFG5, FKS3, GSC2, KRE6, KTR1, LAS21, PST1, ROT2, SCW10, SCW11, SCW4, SKN1, SMK1, SPI1, SUN4, UTR2, YPS1, YPS3 (Supplementary Table S7).

### 3.9. Drug Resistance Gene Analysis

According to the annotation results of the *S. bacillaris* CC-PT4 genome, it can be seen that there are 30 genes related to drug resistance in this strain (Supplementary Table S8).

### 3.10. Pathogenicity Analysis

**Figure 8:**
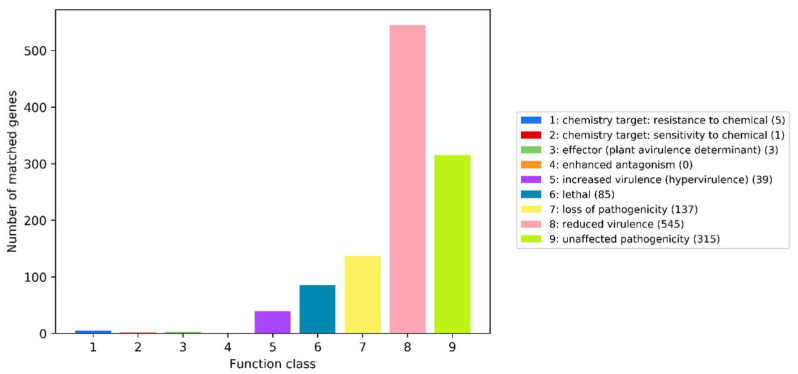
The distribution of pathogen–host interaction (PHI) genes in *Starmerella bacillaris* CC-PT4.

The statistics of the number of PHI phenotype mutation type genes predicted based on the *S. bacillaris* CC-PT4 whole-genome were shown in Figure 8. A total of 1013 genes were annotated through the PHI database, of which the largest number was reduced virulence, with 545 genes, while the number of enhanced antagonism was zero. Through further analysis, it was found that there were 34 genes related to human disease (Table 5).

DFVF is a comprehensive online database of fungal virulence factors. The *S. bacillaris* CC-PT4 whole-genome was compared with the DFVF database and 529 annotation results were obtained. Indeed, 443 of these annotated genes also appeared in the annotation results of the PHI database, and 20 of them were related to human disease (Table 5).

## 4. Discussion

*S. bacillaris* is often isolated from grapes and winemaking environments, improves the flavor of wine, and acts as a biocontrol agent to inhibit fungi [15,41,42]. Our recent study has shown that *S. bacillaris* CC-PT4 also had inhibitory effects on MRSA and was tolerant to harsh environments, such as acids and bile salts [17]. In this study, the whole genome of *S. bacillaris* CC-PT4 was sequenced and analyzed. The results showed that the genome size of yeast was 9.45 Mb, and the GC content was 39.5%. GC content is a feature of microbial taxonomic descriptions [43]. The genome sizes of *S. bacillaris* type strain CBS 9494, *S. bacillaris* FRI751, and *S. bacillaris* PAS13 were 9.3 Mb, 9.3 Mb, and 9.4 Mb, respectively. The GC contents were 39.4%, 39.4%, and 39.45%, respectively [22,44,45]. The genome size and GC content of *S. bacillaris* CC-PT4 were close to these strains, which verified that the strain belonged to *S. bacillaris*.

The completeness of genome assembly was assessed in this study according to the percentage of the number of single-copy genes that are completely aligned in the genome sequence to the total number of single-copy genes. As a result, 63.3% of the genes of this strain could be completely aligned to the BUSCO single-copy gene. This may be due to the lack of data in the database or acceleration of genome evolution of the strain [46]. Research has also shown that the *S. bacillaris* strains isolated in the brewing environment are very diverse at the genetic level and contain a large number of genes of alien origin in the process of evolution [21,47].

The *S. bacillaris* CC-PT4 genome was predicted to contain 4150 protein-coding genes, and several databases were used to annotate these genes. However, according to the annotation by the NCBI nr database, the maximum number of annotated genes corresponding to *Wickerhamiella sorbophila* was 2432; that is, 68.74% of the genes annotated by *S. bacillaris* CC-PT4 have homology with *Wickerhamiella sorbophila* but only nine genes annotated with *S. bacillaris* (only 0.25%). In fact, according to the molecular phylogeny of whole-genome data, it has been proved that the phylogenies of *Starmerella* and *Wickerhamiella* are very close, belonging to an evolutionary branch [48]. In addition, it may be due to the lack of *S. bacillaris* protein data in the NCBI database, resulting in fewer annotations to *S. bacillaris* species. In fact, there were 4740 reference sequences of *Wickerhamiella sorbophila* protein in NCBI RefSeq and only 15 reference sequences of *Starmerella bacillaris* protein (https://www.ncbi.nlm.nih.gov/protein/, accessed on 26 October 2022).

The prediction of CAZy in the *S. bacillaris* CC-PT4 genome found that the highest content was glycosyl transferases (GTs) with 44 genes, followed by glycoside hydrolases (GHs) with 28 genes and without polysaccharide lyases (PLs). GH enzymes have the potential to hydrolyze complex carbohydrates, and GTs are important for surface structures recognized by the host immune system. Higher numbers of glycosyl transferases (GTs) and glycoside hydrolases (GHs) suggest the strain has potential in defense against pathogens and immune stimulation [49]. Moreover, 17 of these CAZy were secreted proteins, of which two are GTs and fifteen GHs (Supplementary Table S9). Among them, scaffold1.t1602 and scaffold2.t307 belong to GH18, which can degrade fungal cell wall. Additionally, scaffold2.t307 was annotated by Swiss-Prot as killer toxin subunits alpha/beta, which can interact with the cell walls of sensitive cell and block growth of them. Killer toxin subunit alpha is a potent chitinase, and the GH18 family also includes chitinases, indicating that scaffold2.t307 was annotated consistently by database CAZy and Swiss-Prot. This proves that scaffold2.t307 has the function of destroying fungal cell wall.

*S. bacillaris* CC-PT4 has been shown to inhibit the growth of methicillin-resistant *Staphylococcus aureus* (MRSA) [17]. In this study, it was found that this strain has a gene encoding lysostaphin (scaffold2.t1859) through annotation of the *S. bacillaris* CC-PT4 genome by Swiss-Prot (Table 4). Moreover, many bacteriostatic substances were also found in the annotation results of the secondary metabolite biosynthesis gene clusters in the *S. bacillaris* CC-PT4 genome (Table 3). The compound predicted by scaffold1.t210 and scaffold1.t749 was ustilagic acid, which has a broad bacteriostatic effect against both bacteria and fungi [50]. Further, scaffold1.t750-annotated compound squalestatin S1 has antifungal effects [51]. The compounds annotated by scaffold1.t212 were either Sch-47554 or Sch-47555. Both compounds also have antifungal effects [52].

It has been shown that *S. bacillaris* CC-PT4 was tolerant of harsh conditions such as temperature, bile salts, and acids [17]. This study discovered the *S. bacillaris* CC-PT4 genome contains several genes that help this strain adapt to harsh conditions. There were seven F1F0-ATPases, two Na(+)/H(+) antiporters, one cation/H(+) antiporter, and multiple proton ATPases and related subunits in the yeast genome (Supplementary Table S6). F1F0-ATPase, cation/H(+) antiporter, and Na(+)/H(+) antiporter have the effect of exporting protons from the cytoplasm and are considered to be the main factors for regulating pH in cells, increasing the resistance of strain to acid [49,53]. Bile salts have toxic effects, mainly destroying the cell membrane and cell wall, inducing DNA damage and oxidative stress [54,55]. Production of bile salt hydrolase is an effective way to cope with the toxicity of bile salts, but bile salt tolerance can also be improved by actively excreting bile salts and expressing some genes that maintain the cell wall, cell membrane, and general stress response [54]. No related genes encoding bile salt hydrolase were found in the *S. bacillaris* CCPT4 genome, but there were genes encoding ATP-dependent bile acid permease (Supplementary Table S6). In addition, there are major facilitator superfamily (MFS) antiporters in the *S. bacillaris* CCPT4 genome (Supplementary Table S8), which can not only expel drugs from cells but also eliminate substances such as bile salts. These genes contribute to *S. bacillaris* CCPT4 resistance to bile salt stress. In addition, the genes involved in oxidative stress resistance were found in the *S. bacillaris* CCPT4 genome (Supplementary Table S6), such as superoxide dismutase. Magnesium transporter, zinc transporter, and metal resistance protein for ionic and heavy metal stress resistance were found in the *S. bacillaris* CCPT4 genome (Supplementary Table S6). A number of heat shock proteins were also identified, indicating the heat tolerance in *S. bacillaris* CCPT4. Moreover, the *S. bacillaris* CCPT4 genome contains genes encoding general stress response protein, DNA repair protein, cell wall integrity and stress response component 4, etc., which help this strain cope with harsh environments.

By BLAST analysis, five genes in the *S. bacillaris* CCPT4 genome encoded flocculation proteins (FLO1, FLO5, FLO9, FLO10, FLO11). However, a gene had sequence identity with multiple fragments of a flocculation protein, or a gene had sequence identity with several flocculation proteins (Figure 7). This is because the sequence fragments in flocculation proteins are repetitive, and the increase or decrease in these repeating units also affects the adhesion properties of flocculation proteins [36]. Additionally, FLO1, FLO5, FLO9, and FLO10 have sequence homology [56]. Flocculation proteins are cell–cell adhesion, and adhesion is the interaction with other foreign surfaces. In the *S. bacillaris* CCPT4 genome, 65 genes associated with adhesion were found.

The safety-related genes of *S. bacillaris* CCPT4 were also analyzed, including drug resistance and pathogenic genes. Based on the annotation results of protein-coding genes, 30 genes related to drug resistance were identified, including ABC multidrug transporter, multidrug resistance protein, MFS transporter, MFS multidrug transporter, and MFS antiporter (Supplementary Table S8). ABC multidrug transporter may increase resistance to azoles. The MFS transporter family is a multidrug efflux system that can transport a variety of structurally unrelated compounds from cells, including cycloheximide and azoles, making strains resistant to many compounds [57,58]. Previous research has also shown that *S. bacillaris* CCPT4 was resistant to fluconazole and itraconazole but sensitive to amphotericin B [17]. Combined with the annotations from the PHI and DFVF databases, some virulence factors may be present in the *S. bacillaris* CCPT4 genome, so this strain should be used with more caution and further studies should be conducted.

## 5. Conclusions

In this study, the whole genome sequence of *S. bacillaris* CC-PT4 was assembled and bioinformatics analyses were performed. The whole genome size of the strain was 9.45 Mb and the GC content was 39.50%. Further, 4150 protein-coding genes were predicted and annotated using several bioinformatics databases. The annotation results of protein-coding genes revealed that many genes were related to adaptation to stress, secondary metabolite, antibacterial function, safety, etc., including two secondary metabolite biosynthesis gene clusters, and two genes encoded killer toxin and one gene encoded lysostaphin. In all, the whole genome sequence of *S. bacillaris* CC-PT4 helps to better understand the characteristics of this strain, which is conducive to mining and application of this strain.

## Figures and Tables

**Table 1:** Sequence identity statistics of the *S. bacillaris* CC-PT4 genome annotated in NCBI nr (top 10).

Query_Name	Hit_Name	Hit_Species	Identity (%)
scaffold1.t310	ALK02041.1	*Starmerella bacillaris*	100
scaffold1.t1149	AGQ04602.1	*Starmerella bacillaris*	100
scaffold1.t1341	AGQ04605.1	*Starmerella bacillaris*	100
scaffold2.t730	AGQ04603.1	*Starmerella bacillaris*	100
scaffold1.t293	AGQ04604.1	*Starmerella bacillaris*	99.93
scaffold1.t322	AGQ04600.1	*Starmerella bacillaris*	99.88
scaffold1.t583	AGQ04601.1	*Starmerella bacillaris*	99.83
scaffold2.t1627	AJE25530.1	*Starmerella bombicola*	98.67
scaffold1.t311	ALK02042.1	*Starmerella bacillaris*	98.60
scaffold4.t1	YP_052724.2	*Starmerella bacillaris*	97

Note: Hit_species, the species corresponding to the annotated gene.

**Table 2:** CAZy analysis statistics.

Type	Number of Genes	Percentage (%)
Glycosyl Transferases	44	1.06
Glycoside Hydrolases	28	0.67
Carbohydrate Esterases	12	0.29
Auxiliary Activities	9	0.22
Carbohydrate-Binding Modules	2	0.05
Polysaccharide Lyases	0	0

**Table 3:** Annotation results of the *S. bacillaris* CC-PT4 secondary metabolic synthesis gene cluster based on MIBIG.

Query_Name	MIBIG Cluster	BLAST Score	Main Product
scaffold1.t210	BGC0001281	253.0	ustilagic acid
scaffold1.t211	BGC0000397	420.0	nostocyclopeptide A2
scaffold1.t212	BGC0000268	527.0	Sch-47554, Sch-47555
scaffold1.t749	BGC0001281	116.0	ustilagic acid
scaffold1.t750	BGC0001839	346.0	squalestatin S1

**Table 4:** Killer toxin and lysostaphin gene in *S. bacillaris* CC-PT4 genome.

Query_Name	Hit_Name	Hit_Description	Database
scaffold1.t1525	O94474	Superkiller protein 3	Swiss-Prot
scaffold2.t307	P09805	Killer toxin subunits alpha/beta	Swiss-Prot
scaffold2.t1859	P10548	Lysostaphin	Swiss-Prot

**Table 5:** *Starmerella bacillaris* CC-PT4 virulence analysis.

Query_Name	PHI ^a^		DFVF ^b^	
	Hit_Name	Identity (%)	Hit_Name	Identity (%)
scaffold1.t37	Q6FVH8	31.45	OPSB_ASPFU	31.78
scaffold1.t92	A4VWD1	27.96		
scaffold1.t160	Q8Y8N0	28.79	F2QYD1_PICP7	26.64
scaffold1.t163	K8DPN9	33.94		
scaffold1.t212	Q8MPM3	50.76		
scaffold1.t352	P08179	32.47		
scaffold1.t675	G8B6Y8	33.33	OPSB_ASPFU	28.89
scaffold1.t729	A0A2R6UD96	38.98		
scaffold1.t922	A5IXN3	35.91		
scaffold1.t977	Q8Y8N0	38.10	DHH1_CRYNV	34.17
scaffold1.t1086	Q8Y8N0	37.43	DHH1_CRYNV	40.71
scaffold1.t1160	C4YFX2	26.13	TUP1_CANAL	26.13
scaffold1.t1414	Q8Y8N0	26.85	MAK5_COCIM	27.98
scaffold1.t1501	C4YFX2	36.00	TUP1_CANAL	36.00
scaffold1.t1597	Q8Y7M8	29.50	C1GMG4_PARBD	28.41
scaffold1.t1605	C4YFX2	25.20	TUP1_CANAL	25.20
scaffold2.t288	C4YFX2	24.77	TUP1_CANAL	24.77
scaffold2.t751	Q8Z4H3	36.23		
scaffold2.t774	Q8Y8N0	36.15	DHH1_CRYNV	32.02
scaffold2.t863	A0A1D8PS71	36.42		
scaffold2.t1078	Q5AC08	34.96	Q5ACC9_CANAL	34.96
scaffold2.t1137	P0DJM0	29.55	B9WMV3_CANDC	34.83
scaffold2.t1188	Q8Y8N0	40.57	DHH1_CRYNV	34.90
scaffold2.t1236	P19786	39.52		
scaffold2.t1305	Q59M00	40.84		
scaffold2.t1501	Q8Y8N0	34.24	DHH1_CRYNV	29.53
scaffold2.t1544	Q59M00	28.57		
scaffold2.t1811	Q8Z4H3	30.47	Q3Y5V5_MAGGR	28.96
scaffold3.t273	C4YFX2	22.91	TUP1_CANAL	22.91
scaffold3.t300	Q8Y6G5	20.72		
scaffold3.t352	A0A2R6UD96	38.92		
scaffold3.t406	Q5A7S7	31.80	Q5A7S7_CANAL	36.16
scaffold3.t454	Q6FVH9	35.71	OPSB_ASPFU	35.88
scaffold3.t461	Q6FVH9	24.89		

^a^: *Starmerella bacillaris* CC-PT4 gene annotation results in PHI associated with human disease; ^b^: *Starmerella bacillaris* CC-PT4 gene was annotated in both DFVF and PHI and was associated with human disease.

## Data Availability

The data presented in this study are available in this published article and its Supplementary Materials.

## Supplementary Materials

The following supporting information can be downloaded at: https://www.mdpi.com/article/10.3390/jof8121255/s1, Table S1: Statistics of genome assembly results, Table S2: Integrity assessment of genome assembly, Table S3: Statistics of repetitive sequences in the *S. bacillaris* CC-PT4 genome, Table S4: Statistics of non-coding RNAs, Table S5: Summary of predicted genes, Table S6: Stress responsive genes in *S. bacillaris* CC-PT4 genome, Table S7: *S. bacillaris* CC-PT4 whole-genome BLAST alignment with flocculin and adhesion proteins, Table S8: Drug resistance related genes in *S. bacillaris* CC-PT4 whole-genome, Table S9: List of carbohydrate-active enzyme genes in secreted proteins.
