# Supplementary material for: Whole-Genome Analysis of Starmerella bacillaris CC-PT4 against MRSA, a Non-Saccharomyces Yeast Isolated from Grape
Source: J Fungi (Basel). 2022 Nov 28;8(12):1255. doi: 10.3390/jof8121255 (PMC9784136; doi:10.3390/jof8121255)
Supplement: Supplementary file 1 [file jof-08-01255-s001.zip › supplementary Table S1-8.pdf]

Supplement Table

Table S1. Statistics of genome assembly results.

Parameter	PT4.scaffold
Min sequence length (bp)	8503
Max sequence length (bp)	4159713
Total sequence number	5
N20	4159713
N20 Number	1
N50	4154241
N50 Number	2
N90	1108139
N90 Number	3
N number	100
N rate	1e-05
Total sequence length (bp)	9451933
GC content %:	39.50
Sequences greater than1 kb	5

N number: number of indeterminate bases, N rate: the ratio of the number of indeterminate bases to the length of the spliced sequence.

Table S2. Integrity assessment of genome assembly.

Property	number	percent(%)
Complete BUSCOs	1353	63.3%
Complete and single-copy BUSCOs	1351	63.2%
Complete and duplicated BUSCOs	2	0.1%
Fragmented BUSCOss	79	3.7%
Missing BUSCOs	705	33.0%
Total BUSCO groups searched	2137	100%

Complete BUSCOs: The number of single-copy genes in complete alignment to BUSCO; Complete and single-copy BUSCOs: Complete alignment, and the alignment result is the number of one copy gene; Complete and duplicated BUSCOs: The number of complete alignments with more than one copy of the gene; Fragmented BUSCOs: The number of partially aligned BUSCO single-copy genes; Missing BUSCOs: The number of unaligned BUSCO single-copy genes; Total BUSCO groups searched: The total number of single-copy genes included in BUSCO.

Table S3. Statistics of repetitive sequences in the *S. bacillaris* CC-PT4 genome.

Type	Element	Number	Length (bp)	Percent(%)
Interspersed repeats	Retroelements	413	34403	0.36
	DNA transposons	249	20222	0.21
	Unclassified:	358	58606	0.62
Tandem repeats	Satellites:	38	3200	0.03
	Simple repeats:	179	14116	0.15
	Low complexity:	69	7478	0.08

Precent (%) :percentage of total genome length.

Table S4. Statistics of non-coding RNAs.

Type	Copy	Avg. Length (bp)	Total Length (bp)	Percent(%)
5S rRNA	1	117.0	117	0.0012
5.8S rRNA	1	145.0	145	0.0015
18S rRNA	1	1753.0	1753	0.0185
28S rRNA	1	3446.0	3446	0.0364
tRNA	123	81.3333	10004	0.1
Other	7	118.0	826	0.0087

Note: Other, other non-coding RNAs except rRNA and tRNA; Precent (%) as a percentage of the total length of the genome.

Table S5. Summary of predicted genes.

Parameter	Value
Total Genes length	6020904
Genes Percentage of genome	63.70%
Total Genes Number	4150
Average gene length	1450.8
Total Exons Number	4266
Average Exons Per Gene	1
Total Exons length	5996642
Exons Percentage Of genome	63.44%
Average Exons length	1405.6
Average Introns length	209.1
Total CDSs length	5996642
CDSs Percentage of genome	63.44%
Average CDS length	1444.9

Table S6. Stress responsive genes in *S. bacillaris* CC-PT4 genome.

Gene ID	Hit_description	Database
pH stress resistance		
scaffold1.t442	F1F0-ATPase	eggNOG
scaffold1.t483	F1F0-ATPase	eggNOG
scaffold1.t707	F1F0-ATPase	eggNOG
scaffold1.t1762	F1F0-ATPase	NCBI nr
scaffold2.t214	F1F0-ATPase	eggNOG
scaffold2.t1250	F1F0-ATPase	eggNOG
scaffold3.t72	F1F0-ATPase	eggNOG
scaffold2.t1363	cation/H(+) antiporter	NCBI nr
scaffold1.t1070	Na(+)/H(+) antiporter	Swiss-prot
scaffold2.t1672	Na(+)/H(+) antiporter	Swiss-prot
scaffold1.t1279	H+-ATPase subunit G	NCBI nr
scaffold3.t246	H+ ATPase	NCBI nr
scaffold1.t1411	V-type proton ATPase subunit H	NCBI nr
scaffold2.t1694	V-type proton ATPase subunit F	Swiss-prot
scaffold2.t17	V-type proton ATPase subunit E	NCBI nr
scaffold1.t447	V-type proton ATPase subunit D	Swiss-prot
scaffold2.t970	V-type proton ATPase subunit D	NCBI nr
scaffold1.t937	V-type proton ATPase subunit C	NCBI nr
scaffold1.t1225	V-type proton ATPase subunit B	NCBI nr
scaffold1.t1229	V-type proton ATPase subunit B	NCBI nr
scaffold1.t1345	V-type proton ATPase subunit A	Swiss-prot
scaffold2.t1594	V-type proton ATPase subunit A	NCBI nr
scaffold1.t1392	V-type proton ATPase catalytic subunit A	Swiss-prot
scaffold1.t402	V-type proton ATPase 16 kDa proteolipid subunit 2	Swiss-prot
scaffold1.t1406	V-type proton ATPase 16 kDa proteolipid subunit	Swiss-prot
scaffold2.t1514	V-type ATPase assembly factor pkr1	Swiss-prot
scaffold2.t1543	V-type proton ATPase 20 kDa proteolipid subunit	Swiss-prot
Bile stress resistance		
scaffold1.t330	ATP-dependent bile acid permease	NCBI nr
Oxidative stress resistance		
scaffold1.t1775	tyrosine-protein phosphatase	eggNOG
scaffold1.t304	Thioredoxin-2	Swiss-prot
scaffold1.t1617	Thioredoxin	eggNOG
scaffold2.t998	Thioredoxin	NCBI nr
scaffold2.t1470	Thioredoxin reductase	Swiss-prot
scaffold2.t1601	Superoxide dismutase [Mu]	NCBI nr

scaffold2.t1659	Superoxide dismutase [Cu-Zn]	NCBI nr
scaffold2.t538	Superoxide dismutase	eggNOG
scaffold2.t1489	Superoxide dismutase	eggNOG
scaffold1.t1139	peroxisomal peroxiredoxin	NCBI nr
scaffold2.t1141	Peroxisomal catalase	NCBI nr
scaffold1.t285	Peroxiredoxin TSA1	NCBI nr
scaffold1.t154	Peroxiredoxin DOT5	NCBI nr
scaffold1.t1606	Peroxiredoxin PRX1	Swiss-prot
scaffold2.t76	Peroxiredoxin PRX1	Swiss-prot
scaffold2.t483	Oxidative stress response two-component system protein SSK1	Swiss-prot
scaffold1.t475	Glutathione peroxidase-like peroxiredoxin HYR1	Swiss-prot
scaffold1.t1363	Glutathione peroxidase-like peroxiredoxin HYR1	Swiss-prot
scaffold2.t1034	Glutathione reductase	eggNOG
Ionic and heavy metal stress resistance		
scaffold2.t1436	Cobalt/magnesium transport protein CorA	Swiss-prot
scaffold1.t1103	Magnesium transporter ALR1	NCBI nr
scaffold2.t1374	Magnesium transporter NIPA4	NCBI nr
scaffold1.t29	Mitochondrial inner membrane magnesium transporter LPE10	NCBI nr
scaffold2.t18	Mitochondrial inner membrane magnesium transporter MRS2	NCBI nr
scaffold2.t339	Mitochondrial magnesium exporter 1	Swiss-prot
scaffold2.t575	magnesium transporter NIPA4	NCBI nr
scaffold1.t64	Zinc transporter Slc39a7	NCBI nr
scaffold1.t679	zinc transporter zrg17	NCBI nr
scaffold2.t667	zinc transporter cis4	NCBI nr
scaffold3.t317	Zinc transporter ZIP9	Swiss-prot
scaffold1.t730	Zinc-regulated transporter 3	Swiss-prot
scaffold1.t452	Zinc-regulated transporter 1	NCBI nr
scaffold1.t1061	Zinc-responsive transcriptional regulator ZAP1	NCBI nr
scaffold2.t460	Mitochondrial zinc maintenance protein 1	NCBI nr
scaffold1.t892	zinc cadmium resistance protein	NCBI nr
scaffold3.t317	ZIP metal ion transporter	eggNOG
scaffold1.t1078	metal ion transporter C17A12.14	NCBI nr
scaffold2.t895	Mitochondrial metal transporter 2	NCBI nr
scaffold2.t246	Metal-binding activator 1	NCBI nr
scaffold1.t260	Metal resistance protein YCF1	Swiss-prot
scaffold1.t1140	Metal resistance protein YCF1	Swiss-prot
scaffold3.t138	Metal resistance protein YCF1	NCBI nr

scaffold1.t81	Metal homeostatis protein BSD2	NCBI nr
scaffold1.t1103	CorA family metal ion transporter	eggNOG
scaffold2.t1436	CorA family metal ion transporter	eggNOG
scaffold2.t633	cell surface metalloreductase	eggNOG
scaffold3.t226	cell surface metalloreductase	eggNOG
Heat stress resistance		
scaffold1.t121	Heat shock protein	NCBI nr
scaffold1.t167	Heat shock protein	eggNOG
scaffold1.t177	Heat shock protein	NCBI nr
scaffold1.t318	Heat shock protein homolog SSE1	Swiss-prot
scaffold1.t1138	Heat shock protein	eggNOG
scaffold1.t1447	Heat shock protein 70 homolog LHS1	Swiss-prot
scaffold2.t242	Heat shock protein 90	NCBI nr
scaffold2.t292	heat shock protein 70	NCBI nr
scaffold2.t422	Heat shock protein 78	NCBI nr
scaffold2.t926	Heat shock protein SSC1	NCBI nr
scaffold2.t1735	Heat shock protein	Swiss-prot
scaffold3.t299	Heat shock protein STI1	NCBI nr
scaffold3.t337	Heat shock protein SSB	NCBI nr
scaffold1.t1195	Protein BOBBER 1	Swiss-prot
scaffold1.t1413	Heat shock transcription factor	Swiss-prot
scaffold2.t1781	heat shock factor protein	NCBI nr
Other stress resistance		
scaffold1.t771	General stress response protein Whi2	eggNOG
scaffold3.t234	Stress response protein ish1	Swiss-prot
scaffold1.t186	DNA mismatch repair protein Msh2	eggNOG
scaffold1.t240	DNA repair protein Rad51	eggNOG
scaffold1.t241	DNA damage response protein	eggNOG
scaffold1.t268	DNA repair and recombination protein pif1	eggNOG
scaffold1.t361	DNA repair and recombination protein	eggNOG
scaffold1.t471	DNA repair helicase	eggNOG
scaffold1.t473	DNA mismatch repair protein Msh6	eggNOG
scaffold1.t839	DNA repair protein	eggNOG
scaffold1.t914	Component of the post-replicative DNA mismatch repair system	eggNOG
scaffold1.t926	DNA repair protein	eggNOG
scaffold1.t964	DNA damage repair protein Rad9	eggNOG
scaffold1.t295	Serine threonine protein kinase	eggNOG
scaffold2.t184	Component of the cytosolic iron-sulfur (Fe S) protein assembly machinery	eggNOG
scaffold2.t1734	stress activated MAP kinase interacting protein	eggNOG
scaffold1.t62	Cell wall integrity and stress response component 4	NCBI nr

scaffold2.t1177	Cell wall integrity and stress response component 1	Swiss-prot

Table S7. *S. bacillaris CC-PT4* whole-genome BLAST alignment with flocculin and adhesion proteins

Query ID	Subject ID	Query Start	Query End	Subject Start	Subject End	Identity (%)
Flocculin genes						
FLO1	scaffold2.t1852	278	1106	961	1734	33.94
FLO1	scaffold2.t1852	277	1084	371	1127	34.41
FLO1	scaffold2.t1852	309	1135	360	1130	33.74
FLO1	scaffold1.t378	135	280	317	449	31.76
FLO5	scaffold2.t1852	255	684	352	752	35.50
FLO5	scaffold2.t1852	273	684	1082	1466	34.71
FLO5	scaffold1.t378	135	282	317	450	30.67
FLO9	scaffold2.t1852	278	907	372	960	33.18
FLO9	scaffold2.t1852	277	907	834	1422	32.86
FLO9	scaffold2.t1859	123	251	53	182	30.77
FLO10	scaffold2.t1859	132	273	46	182	32.24
FLO10	scaffold1.t378	157	294	317	441	30.00
FLO11	scaffold1.t1051	309	877	2383	2992	44.57
FLO11	scaffold1.t1051	305	874	2566	3176	44.25
FLO11	scaffold1.t1051	305	881	3225	3709	47.64
FLO11	scaffold1.t1051	309	913	2746	3429	42.31
FLO11	scaffold1.t1051	313	868	3112	3663	44.80
FLO11	scaffold1.t1051	313	874	3354	3865	47.36
FLO11	scaffold1.t1051	377	874	2194	2704	43.57
FLO11	scaffold1.t1051	456	875	2232	2639	45.16
FLO11	scaffold1.t1051	313	580	3594	3880	42.06
FLO11	scaffold1.t1055	309	862	995	1602	33.54
FLO11	scaffold1.t1055	290	862	1409	2058	32.99
Adhesion genes						
BGL2	scaffold2.t1330	1	312	37	346	63.61
CKA2	scaffold1.t76	17	334	4	321	63.01
CKA2	scaffold1.t627	46	175	43	189	30.20
CKA2	scaffold1.t1040	152	237	714	796	32.56
CKA2	scaffold1.t1572	151	243	201	290	30.53
CKA2	scaffold2.t166	48	190	113	261	30.92
CKA2	scaffold2.t604	144	243	548	646	30.00
CKA2	scaffold3.t82	54	334	25	303	30.82
CKA2	scaffold3.t122	139	216	1403	1483	36.15

CRH1	scaffold2.t502	1	249	7	284	30.18
CRH1	scaffold2.t739	98	431	125	523	32.26
CRH1	scaffold2.t1291	95	282	89	257	30.00
CRH1	scaffold3.t330	40	275	54	322	31.75
CRR1	scaffold2.t502	37	355	31	297	33.54
CRR1	scaffold2.t739	30	417	22	355	31.54
CRR1	scaffold2.t1291	187	309	98	209	31.20
CRR1	scaffold3.t330	37	408	31	346	31.90
CWH41	scaffold1.t791	40	830	11	737	34.19
DCW1	scaffold2.t390	21	450	16	500	43.21
DCW1	scaffold2.t393	27	450	21	454	51.72
DFG5	scaffold2.t390	11	459	1	500	40.87
DFG5	scaffold2.t393	35	459	24	454	46.99
FKS3	scaffold1.t1122	15	1786	163	1911	54.08
FKS3	scaffold2.t651	31	1750	124	1793	39.00
GSC2	scaffold1.t1122	13	1890	5	1908	63.33
GSC2	scaffold2.t651	81	1837	25	1779	38.06
KRE6	scaffold3.t217	134	712	52	645	55.83
KTR1	scaffold1.t80	14	376	8	381	44.97
KTR1	scaffold1.t1224	5	375	1	382	41.33
KTR1	scaffold2.t5	60	377	62	392	50.45
KTR1	scaffold2.t1620	70	388	60	386	59.63
KTR1	scaffold2.t1621	17	385	18	375	52.79
KTR1	scaffold2.t1622	70	359	229	516	53.45
LAS21	scaffold1.t735	15	823	17	718	33.05
LAS21	scaffold2.t610	13	345	21	345	33.91
PST1	scaffold1.t1416	28	362	19	353	37.98
ROT2	scaffold2.t576	8	860	6	832	37.82
SCW10	scaffold3.t136	138	388	111	359	62.15
SCW11	scaffold3.t136	283	537	111	356	34.88
SCW4	scaffold3.t136	135	382	111	356	59.27
SKN1	scaffold3.t217	184	764	75	645	53.48
SMK1	scaffold1.t12	44	347	14	310	33.65
SMK1	scaffold1.t30	127	248	96	207	36.89
SMK1	scaffold1.t90	33	340	24	321	35.81
SMK1	scaffold1.t522	149	263	117	225	32.17
SMK1	scaffold1.t533	38	339	16	321	31.75
SMK1	scaffold1.t594	18	362	33	380	33.98
SMK1	scaffold1.t767	44	243	910	1102	30.29
SMK1	scaffold1.t782	150	243	111	205	36.28
SMK1	scaffold1.t1040	137	245	700	797	36.04
SMK1	scaffold1.t1141	38	358	20	319	40.19
SMK1	scaffold1.t1194	38	291	530	758	30.12
SMK1	scaffold1.t1340	37	239	286	486	32.06

SMK1	scaffold1.t1727	145	264	124	234	32.50
SMK1	scaffold2.t104	38	340	6	290	32.69
SMK1	scaffold2.t135	39	266	23	259	30.92
SMK1	scaffold2.t180	37	239	218	417	32.55
SMK1	scaffold2.t224	150	243	40	122	30.85
SMK1	scaffold2.t727	38	340	7	295	32.90
SMK1	scaffold2.t1021	40	344	362	655	33.33
SMK1	scaffold2.t1144	122	258	194	346	30.13
SMK1	scaffold2.t1264	18	380	1	353	45.48
SMK1	scaffold3.t82	22	373	7	348	30.93
SMK1	scaffold3.t122	118	277	1388	1573	31.58
SMK1	scaffold3.t210	38	252	446	655	32.42
SPI1	scaffold1.t1055	73	107	3086	3120	54.29
SPI1	scaffold1.t1055	73	102	3136	3165	63.33
SPI1	scaffold1.t1599	73	149	35	125	45.06
SPI1	scaffold1.t1637	68	104	375	411	56.76
SPI1	scaffold1.t1637	58	103	57	98	52.17
SPI1	scaffold1.t1637	73	103	172	202	64.52
SPI1	scaffold1.t1637	73	103	224	254	64.52
SPI1	scaffold1.t1637	73	103	328	358	64.52
SPI1	scaffold1.t1637	73	103	120	150	64.52
SPI1	scaffold1.t1637	73	103	276	306	64.52
SPI1	scaffold2.t264	73	114	671	712	59.52
SPI1	scaffold2.t264	76	110	768	802	48.57
SPI1	scaffold2.t1247	62	115	177	230	57.41
SPI1	scaffold2.t1273	68	106	22	60	58.97
SPI1	scaffold2.t1692	15	112	35	131	46.23
SPI1	scaffold3.t372	68	136	75	142	53.62
SUN4	scaffold2.t1251	18	421	7	420	51.20
UTR2	scaffold2.t502	33	345	31	339	42.41
UTR2	scaffold2.t739	33	427	30	422	39.45
UTR2	scaffold3.t330	17	398	14	389	40.00
YPS1	scaffold1.t37	82	505	50	423	30.46
YPS1	scaffold1.t675	64	545	33	472	30.83
YPS1	scaffold2.t1447	192	430	83	299	30.12
YPS1	scaffold3.t454	63	505	26	433	33.63
YPS3	scaffold1.t37	62	424	50	423	34.90
YPS3	scaffold1.t675	8	470	1	476	31.29
YPS3	scaffold2.t1078	56	413	82	431	31.48
YPS3	scaffold3.t454	23	427	30	436	37.12

Table S8. Drug resistance related genes in *S. bacillaris* CC-PT4 whole-genome

Gene ID	Hit_description	Database
scaffold2.t1811	ABC multidrug transporter mdr2	Swiss-prot
scaffold2.t409	Multidrug resistance protein	eggNOG
scaffold2.t1138	Multidrug resistance protein fnx1	Swiss-prot
scaffold2.t1331	Multidrug resistance protein	NCBI nr
scaffold1.t260	Multiple drug resistance-associated protein-like transporter 1	NCBI nr
scaffold1.t327	MFS transporter	eggNOG
scaffold2.t347	MFS transporter	eggNOG
scaffold2.t348	MFS transporter	eggNOG
scaffold1.t311	MFS multidrug transporter mdrA	Swiss-prot
scaffold1.t310	MFS multidrug transporter mdrA	Swiss-prot
scaffold1.t1369	MFS multidrug transporter FLU1	Swiss-prot
scaffold1.t1746	MFS multidrug transporter FLU1	Swiss-prot
scaffold1.t497	MFS antiporter QDR2	Swiss-prot
scaffold2.t237	MFS transporter asaE	Swiss-prot
scaffold2.t663	MFS transporter asaE	Swiss-prot
scaffold2.t1538	MFS transporter asaE	Swiss-prot
scaffold2.t302	MFS transporter L2	Swiss-prot
scaffold2.t303	MFS transporter L2	Swiss-prot
scaffold1.t574	MFS transporter PfmaC	Swiss-prot
scaffold1.t827	MFS transporter PfmaC	Swiss-prot
scaffold2.t103	MFS general substrate transporter	NCBI nr
scaffold1.t942	MFS phospholipid transporter (Git1)	eggNOG
scaffold2.t1422	MFS phosphate transporter	eggNOG
scaffold1.t262	Uncharacterized MFS-type transporter C947.06c	Swiss-prot
scaffold1.t261	Uncharacterized MFS-type transporter C947.06c	Swiss-prot
scaffold1.t605	Uncharacterized MFS-type transporter C1271.10c	Swiss-prot
scaffold1.t661	Uncharacterized MFS-type transporter C1271.10c	Swiss-prot
scaffold3.t260	Uncharacterized MFS-type transporter C947.06c	Swiss-prot
scaffold3.t261	Uncharacterized MFS-type transporter C947.06c	Swiss-prot
scaffold3.t262	Uncharacterized MFS-type transporter C947.06c	Swiss-prot
